# Stress System Activation in Children and Adolescents With Autism Spectrum Disorder

**DOI:** 10.3389/fnins.2021.756628

**Published:** 2022-01-13

**Authors:** Gerasimos Makris, Agorastos Agorastos, George P. Chrousos, Panagiota Pervanidou

**Affiliations:** ^1^Unit of Developmental and Behavioral Pediatrics, First Department of Pediatrics, “Aghia Sophia” Children’s Hospital, School of Medicine, National and Kapodistrian University of Athens, Athens, Greece; ^2^Department of Psychiatry II, Division of Neurosciences, Faculty of Health Sciences, School of Medicine, Aristotle University of Thessaloniki, Thessaloniki, Greece; ^3^University Research Institute of Maternal and Child Health and Precision Medicine, National and Kapodistrian University of Athens, Athens, Greece

**Keywords:** children, adolescents and youth, stress, cortisol, catecholamines, HPA axis, autonomic nervous system, autism spectrum disorder

## Abstract

The mission of the human stress system is the maintenance of homeostasis in the presence of real or perceived, acute or chronic stressors. The hypothalamic–pituitary–adrenal (HPA) axis and the autonomic nervous system (ANS) are the stress system-related neuroendocrine pathways. There is abundant evidence that children and adolescents with autism spectrum disorder (ASD) may exhibit atypical function within the HPA axis and the ANS both at the resting state and during the presence of social and/or non-social stressors. The aim of this review is to provide an up-to-date summary of the findings regarding stress system alterations in children and adolescents with ASD. We focus on the variations of stress hormones circadian rhythms, specifically cortisol and alpha-amylase (i.e., a surrogate index of epinephrine/norepinephrine secretion), and on the alterations of stress system responsivity to different stressors. Also, we present imaging and immunological findings that have been associated with stress system dysregulation in children and adolescents with ASD. Finally, we review the pivotal role of HPA axis-ANS coordination, the developmental trajectory of the stress system in ASD, and the possible role of early life stress in the dysregulation of the stress system demonstrated in children and adolescents with ASD. This synthesis will hopefully provide researchers with a foundation for an integrated approach to future research into stress system variations in children and adolescents with ASD.

## Introduction

Autism spectrum disorder (ASD) is a heterogeneous group of neurodevelopmental disorders, which emerge early in childhood and are characterized by deficits in social interaction, verbal and non-verbal communication, and by restricted, repetitive patterns of behavior, interests, or activities (American Psychiatric Association [APA], 2013). For 2016, the Center for Disease Control estimated that the prevalence of ASD in the United States was 1 in 54 children aged 8 years and that 4.3 times as many boys as girls were diagnosed with this condition (Maenner et al., 2020). From a clinical perspective, children with ASD struggle to adapt to changes in everyday life and to stressful or perceived as threatening stimuli. Given that a stressor is a perturbing stimulus that disrupts homeostasis, various predictable and unpredictable environmental stimuli of everyday life, as well as psychological conditions that affect emotion and result in fear, anxiety, anger, or frustration, may result in activation of the stress system. Several psychopathological conditions, such as post-traumatic stress disorder (PTSD), depression, panic anxiety, generalized anxiety disorder, obsessive-compulsive disorder, and schizophrenia have been consistently associated with alterations of the stress system (i.e., hyper- or/and hypo-activation) (Luby et al., 2003; Pervanidou, 2008; Faravelli, 2012; Cullen et al., 2014; Dieleman et al., 2015; Funke et al., 2017). The direction of the stress system imbalance (i.e., hyper- or hypo-activation) may depend on genetic and epigenetic factors, previous stress history, and the nature, chronicity, severity, and predictability of the environmental stressors (Agorastos et al., 2019b).

In addition, children and adolescents with neurodevelopmental disorders may exhibit alterations in the diurnal rhythm and/or atypical responses of stress hormones, such as cortisol, to environmental stressors (Anesiadou et al., 2021). It has been suggested that alterations in the functioning of the stress system may be at least partially responsible for the difficulty of ASD children in tolerating novel, otherwise benign environmental stressors (Zinke et al., 2010). On the other hand, core symptoms of ASD, such as sensory deficits, may contribute to stress system dysregulation (Kushki et al., 2013). Moreover, alterations in the regulation of stress system components may account at least partially for several comorbid conditions that often accompany ASD, such as depression and anxiety disorders amongst others (Hollocks et al., 2014; Bitsika et al., 2015, 2018; Sharpley et al., 2016; Muscatello et al., 2021). In fact, excessive and prolonged activation of the stress system might lead to increased and prolonged production of stress system mediators, such as corticotropin-releasing hormone (CRH), cortisol, and catecholamines, which could explain many of the long-term psychopathological complications of chronic stress (Agorastos et al., 2018). Of note, early life stress (ELS) is considered a potent developmental risk factor, likely acting through a number of mediating mechanisms (Kessler et al., 2010; Scott et al., 2011; Agorastos et al., 2019b; Pervanidou et al., 2020). These mechanisms include alterations in the offspring’s development of several brain regions (Charil et al., 2010; VanTieghem and Tottenham, 2017), maternal and fetal immune dysregulation (Beversdorf et al., 2018; Hantsoo et al., 2019; Makris et al., 2021), and epigenetic alterations in the offspring (Bale et al., 2010; Bludau et al., 2019). Therefore, fetal biological systems, including the central nervous system (CNS), the autonomic nervous system (ANS), and neuroendocrine [e.g., the hypothalamic-pituitary-adrenal (HPA) axis], cardiovascular, and immune systems may adapt to prenatal and early postnatal environmental influences leading to subsequent perturbations during development (Charil et al., 2010).

The aim of this review is to provide an up-to-date summary of the findings regarding stress system alterations in children and adolescents with ASD. We focus on the available evidence regarding both variations in the circadian rhythms of stress hormones and alterations in the responsiveness of the stress system to stressors. We also attempt to cast light on the most acknowledged stress system-related neuroendocrine pathways (i.e., the HPA axis and the ANS) and their role in the pathophysiology of ASD. We also review imaging and immunological findings associated with stress system dysregulation in children and adolescents with ASD. Finally, we discuss the pivotal role of HPA axis and ANS coordination during stress system activation, the developmental trajectory of the stress system in ASD, and the possible role of ELS in the dysregulation of the stress system demonstrated in children and adolescents with ASD.

## Stress System Components

The human stress system consists of central and peripheral components, which together serve to help maintain resting and stress-related homeostasis (Chrousos and Gold, 1992). These components drive adaptive behavioral and physical responses to stressors *via* a plethora of mediators, including neuropeptides, neurotransmitters, and steroid hormones. The central effectors of the stress system comprise: (i) the neuropeptide CRH secreted by CRH neurons. The highest concentration of CRH neurons is present in the hypothalamic paraventricular nuclei (PVN). CRH is also secreted by neurons present in several brain regions [i.e., the bed nucleus of the stria terminalis, the central nucleus of the amygdala, the locus caeruleus (LC), the cerebral cortex, and the cerebellum]. Additionally, CRH has been found in chromaffin cells of the adrenal medulla and in the sympathetic ganglia, as well as in peripheral organs and tissues, including the skin, the immune system, and the gastrointestinal tract (Aguilera and Liu, 2012); (ii) the neuropeptide arginine-vasopressin (AVP) also produced by PVN neurons; (iii) the neuromodulators β-endorphin and α-melanocyte-stimulating hormone (MSH) produced in the hypothalamic arcuate nucleus; and (iv) the neurotransmitter norepinephrine produced in the brainstem’s LC and in the noradrenergic (NE) cell groups of the medulla oblongata (LC/NE system). The principal peripheral effectors of the stress system comprise: (i) the catecholamines norepinephrine and epinephrine, regulated by the ANS, including the sympathetic (SNS) and the sympathoadrenomedullary (SAM) nervous systems, and the parasympathetic nervous system (PNS); and (ii) the glucocorticoids (GC) regulated by the HPA axis (Benarroch, 1993; Herman and Cullinan, 1997; Chrousos, 2009; Agorastos et al., 2018).

The HPA axis and the ANS components of the stress system are closely interconnected and functionally complementary to each other (Agorastos et al., 2018). The central ANS is activated promptly after stressor exposure and is immediately followed by an increase of catecholamine secretion in the periphery of the organism. The HPA axis activation then follows with a small time delay (Chrousos and Pervanidou, 2014). Initially, CRH is released into the hypophyseal portal vessels, inducing the synthesis and release of adrenocorticotropin hormone (ACTH) from the anterior pituitary gland into the systemic circulation. Subsequently, ACTH stimulates the release of GC, such as cortisol in humans, from the adrenal cortex (Charmandari et al., 2005). The stress response is regulated in a major fashion by negative feedback exerted by circulating GC through binding to glucocorticoid receptors (GRs) (Smith and Vale, 2006). On the other hand, circulating GC potentiate several sympathetically mediated effects, such as peripheral vasoconstriction. Of note, the adrenal cortex is directly innervated by the ANS, which can augment GC release (Ulrich-Lai and Herman, 2009). Importantly, basal levels of circulating GC show a pronounced difference across the day, with the circadian peak occurring in the early morning in diurnal and in the early night in nocturnal animals (Chrousos, 1998). It is widely accepted that the circadian rhythmic release of GC and their tropic hormone ACTH are under the direct control of the central pacemaker in the suprachiasmatic nucleus (SCN) of the hypothalamus. The SCN drives the GC circadian rhythm both by modulating the HPA axis and *via* sympathetic splanchnic innervation of the adrenal gland (Dickmeis, 2009; Dickmeis et al., 2013). Nevertheless, a growing body of evidence points to the important role of local oscillators found in peripheral tissues, including the adrenal gland, in maintaining the rhythm by controlling the capacity and responsiveness of adrenal GC secretion, and the biosynthesis of ACTH (Son et al., 2018).

## Hypothalamic–Pituitary–Adrenal Axis Alterations in Children and Adolescents With Autism Spectrum Disorder

There is a large body of published data regarding the dysregulation of the HPA axis in children and adolescents with ASD. Cortisol levels in blood, saliva, and/or urine have been extensively used as an index of the HPA axis activity. Results of measurements obtained with these methods have been correlated with each other, although the fractions of free or unbound vs. protein-bound cortisol differ between them (Taylor and Corbett, 2014). The highest correlation has been found between salivary and total serum cortisol levels (80%) (Estrada-Y-Martin and Orlander, 2011).

### Cortisol Circadian Rhythmicity

Findings regarding the overall diurnal rhythm or other aspects of cortisol measurements, such as the cortisol awakening response (CAR), day cortisol decline (slope), or intra- and inter-individual cortisol level variability, are quite diverse in children and adolescents with ASD. For example, regarding CAR (i.e., the plasma cortisol concentration 30 min after awaking), a study that assessed salivary cortisol levels in adolescent males (aged 11–16 years) with Asperger syndrome (AS) reported a blunted CAR in the clinical sample compared to typically developing (TD) children (Brosnan et al., 2009). Also, a lower morning basal cortisol was reported in both saliva and plasma samples of children with ASD in comparison to age- and sex-matched TD children (Marinović-Ćurin et al., 2008; Hamza et al., 2010). However, the majority of studies that investigated salivary cortisol levels in prepubescent children with ASD have shown a similar CAR between the clinical groups and TD children, suggesting that variations in CAR may be present later in adolescence (Zinke et al., 2010; Corbett and Schupp, 2014; Tomarken et al., 2015; Anesiadou et al., 2021). Interestingly, lower diurnal salivary cortisol has been associated with higher rates of repetitive behaviors in a sample of children with ASD aged between 3 and 9 years (Gabriels et al., 2013). This finding suggests that repetitive behaviors might serve a functional, self-soothing role in ASD children as indicated by reduced HPA axis activity reflected in lower diurnal salivary cortisol. Another possibility is that the HPA axis is down-regulated as a result of prolonged GC system activation associated with daily experiences of individuals with ASD (Gabriels et al., 2013).

As far as it regards the cortisol diurnal decline, no significant differences have been found between adolescent males with ASD and TD controls (Brosnan et al., 2009). Nevertheless, a more shallow slope from morning to evening has been demonstrated in male children (aged 6–12 years) diagnosed with ASD and in children (aged 7–16 years) with high-functioning ASD in comparison to TD children (Corbett et al., 2009; Tomarken et al., 2015). In addition, youth with ASD (ages 7–17 years) have shown higher evening cortisol and a blunted diurnal slope relative to TD peers (Muscatello and Corbett, 2018). In the same line, higher evening salivary cortisol values have been found in prepubescent children with ASD than TD children and have been associated with changes in routine or schedule during the day (Corbett et al., 2008, 2009; Tomarken et al., 2015). Interestingly, a recent study demonstrated that adolescents with ASD (aged 13–17 years) had significantly elevated cortisol levels compared to younger ASD children (aged 7–12 years) and that pubertal development and age were significant predictors of evening cortisol level (Muscatello and Corbett, 2018). These findings suggest that elevated evening cortisol, which may reflect the cumulative effects of stress throughout the day, persists across development in youth with ASD (Muscatello and Corbett, 2018). However, several studies have shown no differences in the evening cortisol measurements between ASD and TD children (Richdale and Prior, 1992; Marinović-Ćurin et al., 2008; Kidd et al., 2013; Anesiadou et al., 2021).

The most consistent finding across several studies regarding HPA axis regulation in children and adolescents with ASD is the higher between-subject and within-subject basal cortisol variability compared to controls (Taylor and Corbett, 2014). Precisely, preschool-aged children (2–5.5 years old) with ASD have shown greater within-subject variability of daytime salivary cortisol patterns compared to TD controls (Kidd et al., 2013). Also, the results suggested that there was an increase in cortisol levels when comparing clinical groups stratified by IQ as an indicator of the functional level (Kidd et al., 2013). In addition, older prepubescent ASD children had greater between-subject and within-subject variability in daily cortisol profiles than their TD counterparts (Corbett et al., 2006, 2008, 2009).

### Hypothalamic–Pituitary–Adrenal Axis Responsivity

Studies that examine the responsiveness of the HPA axis by measuring cortisol in ASD children and adolescents suggested hyper- or hypo-responsivity depending on the nature of the stressor. For example, two studies reported a slower cortisol response to ACTH stimulation among ASD children indicated by lower plasma cortisol levels 60 and 90 min post-infusion compared to controls, whereas no differences were found between the groups after 120 min (Marinović-Ćurin et al., 2008; Hamza et al., 2010). Contrarily, higher rates of non-suppression of cortisol in the dexamethasone suppression test (DST) were shown in children with ASD than TD children, suggesting that the negative feedback mechanism of the HPA-axis may be disturbed in ASD children (Jensen et al., 1985; Hoshino et al., 1987).

Concerning non-social environmental stressors, i.e., stressors that do not explicitly manipulate the social environment, a lower rise of cortisol was found in children with pervasive developmental disorder – not otherwise specified (PDD-NOS) than in controls after riding a stationary bike for 10 min; however, a subsequent study found no differences in HPA axis responsiveness after the same physical activity (Jansen et al., 1999, 2003). Moreover, the exposure to a mock MRI resulted in a salivary cortisol increase in ASD children (mean age = 8.5) compared to TD controls, who, in contrast, showed no response or even a reduction in cortisol after being placed in the mock MRI (Corbett et al., 2006). However, subsequent studies did not report alterations in children with ASD compared to TD children regarding cortisol response following exposure to a novel physical stressor (i.e., a mock MRI) (Corbett et al., 2008, 2009). The only study that used the blood draw procedure as a physical stressor demonstrated a higher rise and prolonged response of salivary cortisol in children with ASD (3–10 years old) than TD controls (Spratt et al., 2012).

Studies examining the HPA axis responsivity to psychosocial stressors in children and adolescents with ASD have produced conflicting findings. The majority of studies using public speaking tasks as stressors, such as the Trier Social Stress Test for Children (TSST-C; Kirschbaum et al., 1993), demonstrated a blunted or relatively stable cortisol response in children and adolescents with ASD or high-functioning ASD relative to TD controls indicating that the TSST-C may not reliably activate the HPA axis in individuals with ASD (Jansen et al., 2003; Lanni et al., 2012; Levine et al., 2012; Hollocks et al., 2014; Edmiston et al., 2017a). Conversely, relatively benign social interactions comprising peer interaction paradigms have been found to result in enhanced HPA axis responsivity in children with ASD than TD children (Corbett et al., 2010, 2012; Schupp et al., 2013). Finally, a recent study found similar mean percentage changes of salivary cortisol after an academic performance test and a moral cognition task between school-aged children with high-functioning ASD and TD controls (Anesiadou et al., 2021).

Importantly, several factors may contribute to the observed increased variability of HPA axis responsiveness to stressors that manipulate the social environment of ASD children. For example, children with ASD exhibit higher cortisol levels with increasing age and level of social engagement during a naturalistic playground peer interaction paradigm (Corbett et al., 2010; Schupp et al., 2013). In accordance, a recent study that assessed salivary cortisol response to TSST-C in a large sample of early adolescents with ASD (*n* = 138; median age = 11.25 years) compared to TD children (*n* = 103; median age = 11.67 years) demonstrated a blunted cortisol response and a faster return to basal cortisol levels in youth with ASD (Corbett et al., 2021). Furthermore, it was shown that age contributed to an increase in cortisol response to a social evaluative threat during early adolescence in ASD children (Corbett et al., 2021). T he authors suggested that the age-related increase in cortisol responsivity might be attributed to the heightened importance of peers, the increased awareness of the social appraisal of the situation, and the socio-cognitive development taking place during the adolescent transition (Corbett et al., 2021). Moreover, a study of the cortisol response to the Strange Situation Procedure paradigm (SSP; Ainsworth et al., 1978) found that in 2-year-old children with ASD, cortisol responsivity to separation decreased with the presence of more autistic symptoms (Naber et al., 2007).

## Autonomic Nervous System Alterations in Children and Adolescents With Autism Spectrum Disorder

Studies of ANS indices, such as electrodermal activity (EDA) (i.e., a measure of sympathetic response), respiratory sinus arrhythmia (RSA) (i.e., a measure of parasympathetic activity), and short-term heart rate variability (HRV) (i.e., a measure of vagal tone which reflects PNS activity), have shown divergent findings regarding resting state autonomic functioning and autonomic responsiveness to several stress-inducing tasks in children and adolescents with ASD.

### Autonomic Nervous System Resting State Measures

A study comprising children with high-functioning ASD (8–12 years old) showed no differences in EDA or vagal tone at resting state, during the TSST-C, or post-TSST-C in comparison to the TD group (Levine et al., 2012). In contrast, a study revealed baseline sympathetic underactivity in high-functioning ASD children (ages 7–15 years) indexed by lower EDA than TD controls (Bujnakova et al., 2016). Furthermore, no basal alterations of the parasympathetic tone, as measured by RSA, were found in children (ages 8–18 years) and toddlers (aged from 29 to 42 months) with ASD compared to TD controls (Watson et al., 2012; Kushki et al., 2014). On the contrary, resting cardiac vagal tone and cardiac sensitivity to baroreflex were significantly lower and associated with a significant elevation of heart rate (HR), mean arterial blood pressure, and diastolic blood pressure in children with ASD compared to TD controls (Ming et al., 2005). In addition, several studies reported lower amplitude of RSA and/or faster HR at the baseline or resting state in children and adolescents with ASD than controls (Van Hecke et al., 2009; Bal et al., 2010; Neuhaus et al., 2014, 2016; Edmiston et al., 2016). These findings show lower overall vagal regulation of HR in children and adolescents with ASD and have been suggested as indicative of lower resting PNS activity accompanied by a higher SNS activity in ASD. Interestingly, higher baseline RSA amplitudes in ASD children were associated with better social behavior and receptive language abilities (Patriquin et al., 2013). Additionally, baseline RSA and RSA reactivity predicted restricted repetitive behavior severity in a sample of ASD children (ages 5–10 years), further supporting that low baseline cardiac vagal control predicts less adaptive behaviors in children with ASD (Condy et al., 2017). Recently, reduced tonic HRV, which is a measure of the regulatory capacity of the ANS, was found in children with ASD relative to TD children and it was associated with atypical attentional responsivity to sensory stimulation (Lory et al., 2020).

### Autonomic Nervous System Responsivity

A study of the ANS response to a cognitive task (i.e., Color Stroop Task; Stroop, 1935) demonstrated an elevated HR at baseline and during stress conditions and an elevated but blunted phasic EDA at baseline and during a stress test, respectively, in children with ASD (ages 8–15 years) compared to TD children (Kushki et al., 2013). These findings were interpreted as indicative of a hyper-sympathetic state insufficiently attenuated by parasympathetic influences (Kushki et al., 2013). In a subsequent study, the same group of authors found a marginally elevated basal HR and a blunted HR response to a social anxiety task in the ASD group relative to TD children (Kushki et al., 2014). These findings were suggested to be a manifestation of response saturation due to basal hyperarousal (Kushki et al., 2014). In the same study, children of the ASD group showed increased RSA reactivity to a task of social cognition vs. TD children, suggesting that it may reflect a compensatory mechanism applied by the ASD group. Of note, in this study, medication effects accounted for a marginal difference in the outcomes (Kushki et al., 2014).

Another study that assessed the ANS response in adolescent males with ASD (ages 12–18 years) to a social evaluative threat *via* RSA measurements, found that ASD adolescents had lower RSA values than TD controls, both at baseline and during the TSST (Edmiston et al., 2016). Also, it was demonstrated that less change in RSA from baseline during the TSST correlated with greater severity of social problems in the ASD group (Edmiston et al., 2016). Similarly, children with ASD (ages 8–12 years) exhibited decreased RSA, namely lower PNS activity, than TD controls, while viewing an unfamiliar person stimulus. Besides, higher baseline RSA was related to higher levels of social skills, such as faster emotion recognition, in ASD children (Van Hecke et al., 2009; Bal et al., 2010). Furthermore, adolescent males diagnosed with ASD (ages 12–17 years) had reduced SNS activation relative to TD controls, as indicated by the lower change in the pre-ejection period (PEP) (i.e., the interval from stimulation of the left heart ventricle to the opening of the aortic valve) at the onset of a modified version of the TSST (Edmiston et al., 2017b). The authors suggested that the reduced arousal in response to social cues may lead to reduced attention to social stimuli and subsequently to the altered social behaviors characterizing ASD (Edmiston et al., 2017b). Nevertheless, to date, findings regarding the associations between ANS regulation and social skills in children and adolescents with ASD converge on the fact that better skills and fewer difficulties are associated with sympathetic withdrawal and parasympathetic increase during social interaction (Neuhaus et al., 2016; Patriquin et al., 2019).

### Salivary Alpha-Amylase

Salivary alpha-amylase (sAA), a putative surrogate of norepinephrine, has been extensively used as a non-invasive indirect index of SNS activity (Nater and Rohleder, 2009). Alpha-amylase increases with stress and reaches the peak levels approximately 5 min post-stressor. Also, it follows a diurnal profile characterized by a decrease after awaking followed by a progressive increase during the day (Nater et al., 2005, 2006). Lower afternoon levels of sAA have been found to significantly distinguish ASD children aged 20–72 months from age-matched children with Down syndrome and TD controls (Anderson et al., 2013). Additionally, little sAA diurnal variation was observed in the ASD group (ages 33–79 months) relative to TD controls, who showed a significant linear increase of sAA levels throughout the day (Anderson et al., 2013). Moreover, a study found greater within-subject variability in preschool-aged children (2–5.5 years old) with ASD than TD controls (Kidd et al., 2013). Also, sAA secretion levels were approximately 1.5-fold higher when IQ was lower (Kidd et al., 2013). Finally, a recent study demonstrated lower diurnal sAA secretion in unmedicated school-aged children with high-functioning ASD (i.e., IQ > 70) than TD controls (Anesiadou et al., 2021). Nevertheless, given that sAA levels might also reflect parasympathetic activity and that these studies did not include concurrent data regarding PNS activity, it is uncertain whether lower sAA levels are directly associated with SNS hypoactivity or PNS hyperactivity in ASD children (Bosch et al., 2011; Anesiadou et al., 2021).

## Hypothalamic–Pituitary–Adrenal Axis and Autonomic Nervous System Coordination

The HPA axis and the ANS are interrelated components of an internal neurohormonal regulatory system (central autonomic network, CAN) (Benarroch, 1993; Thayer and Lane, 2000). The CAN integrates high-order autonomic control while mediating emotional responses through hypothalamic-brainstem pathways (Thayer and Lane, 2000). For example, the hindbrain nucleus of the solitary tract (NTS) plays a prominent role in the regulation of HPA axis responses to acute or chronic stressors (Herman, 2018). Thus, catecholaminergic NTS systems enhance HPA axis responses to systemic stressors and increase stress reactivity by augmenting adrenal sensitivity to ACTH under conditions of chronic stress (Herman, 2018). Moreover, at least a proportion of the non-catecholaminergic NTS neurons has been found to influence PVN responses, suggesting connections to both interactive pathways and providing descending information, likely *via* descending limbic projections from regions such as the medial prefrontal cortex (PFC) and the amygdala (Benarroch, 1993; Herman, 2018). Thus, dysregulation of the CAN may affect downstream autonomic core centers and alter both peripheral ANS and HPA axis activity and responsivity. Recently, HPA axis stimulation was associated with a reduced vagal tone, while HPA axis suppression had no distinct effect on autonomic activity in healthy subjects, suggesting that the PNS may play a prevailing role in the interplay between the ANS and the HPA axis (Agorastos et al., 2019a). Accordingly, a recent study in TD children (ages 11–12 years) demonstrated that differences in the degree of HPA axis-ANS coordination in response to stress predicted within-person differences in both externalizing and internalizing behaviors (Chen et al., 2020). Most importantly, stress reactivities of cortisol and sAA on their own were not linked to behavior problems (Chen et al., 2020). The authors proposed that among children with behavioral problems, the stress response may be characterized by the inability of turning off the ANS response at high levels of HPA axis activity when cortisol’s stimulating effects might be diminished (Chen et al., 2020).

Of note, the majority of data comprising ANS biomarkers in ASD children and adolescents generally converge on the presence of ANS hyper-arousal related to sympathetic overactivity, parasympathetic underactivity, or atypical interaction of both systems in ASD (Toichi and Kamio, 2003; Cheshire, 2012). Interestingly, a study that examined HPA axis and ANS physiological responses in ASD children (ages 10–13 years) compared to TD controls showed that the autonomic balance between PNS and SNS, precisely increased PNS combined with decreased SNS activity, was associated with the lowest levels of depressive symptoms in the ASD group (Muscatello et al., 2021). Also, this group did not differ on any of the physiological variables nor systems singularly associated with depressive symptoms (Muscatello et al., 2021). These findings suggest that future ASD studies should concurrently examine both HPA axis and the ANS, rather than their singular, isolated functions, to further elucidate possible alterations in this complex multisystem interplay among children and adolescents with ASD.

## Imaging Findings

Limbic circuits connecting the hippocampus, the amygdala, and the PFC play a pivotal role in the induction of neuroendocrine and behavioral stress responses (Fuchs and Flügge, 2003). The amygdala and the hippocampus normally regulate the hypothalamic activity, but chronic stress may impair the feedback-control mechanisms leading to long-term persistent or repetitive stimulation of the stress system (Herman et al., 2005). Several studies have shown aberrant amygdala and hippocampal development in individuals with ASD vs. neurotypical controls (Sparks et al., 2002; Schumann et al., 2004; Nordahl et al., 2012; Avino et al., 2018; Reinhardt et al., 2020; Richards et al., 2020). Functional alterations in areas, such as the hippocampus, PFC, and amygdala may be responsible for stress system alterations seen in ASD (Schultz, 2005).

Moreover, it is well established that stress causes structural and functional brain changes, mainly during critical periods of brain development (Agorastos et al., 2019b). Of note, many of these influences occur epigenetically in a sex-dependent manner (McEwen et al., 2016). ELS has been associated with reduced volume of hippocampus in adulthood, as well as reduced volume of corpus callosum, insula, dorsolateral PFC, orbitofrontal cortex (OFC), anterior cingulate gyrus, and caudate nucleus (Teicher et al., 2016). Moreover, converging evidence supports the association of ELS with amygdala hyper-responsiveness, particularly to negative-emotion-related stimuli, and with diminished connectivity between PFC and the amygdala (Dannlowski et al., 2012, 2013). The amygdala has been extensively implicated in emotional processes, such as emotion inhibition and regulation, implicit emotional learning, emotional modulation of memory, emotion’s influences on attention and perception, and social behavior (Phelps and LeDoux, 2005). Interestingly, altered functional connectivity between the amygdala and PFC has been shown in children with ASD, which may be the result of ELS or chronic stress influences (Rudie et al., 2012; Liu et al., 2020). In addition, the reduced top-down regulation of the amygdala by the PFC may lead to emotion dysregulation and prolonged stress response to social stimuli in ASD, given that the PFC is involved in social and cognitive appraisal of a stressor (Schultz, 2005; Kennedy and Courchesne, 2008; Corbett et al., 2012; Ibrahim et al., 2019). Several studies have reported reduced amygdala coupling with the ventrolateral and ventromedial PFC during emotional face perception tasks in children and adolescents with ASD (Monk et al., 2010; Swartz et al., 2013; Pitskel et al., 2014; Von Dem Hagen et al., 2014). Accordingly, it has been shown that heightened amygdala and diminished medial PFC activities, in combination with elevated circulating corticosterone levels in animal models play a certain role in the negative mnemonic impacts of uncontrollable stress (Kim and Kim, 2019). Furthermore, the growing dominance of amygdala activity over the hippocampus during and even after chronic stress in animal models has been suggested to contribute to emotional symptoms, alongside cognitive impairment, in stress-related psychiatric disorders (Ghosh et al., 2013).

## Immune System and Inflammation

There is a bidirectional neuroimmunoendocrine interaction between the central and peripheral stress system components and the immune axis (Chrousos, 1995; Cain and Cidlowski, 2017). For example, acute stress influences circulating inflammatory markers, such as pro-inflammatory cytokines (Steptoe et al., 2007). Subsequently, pro-inflammatory cytokines stimulate the secretion of GC, which in turn promote the resolution of the inflammatory response (Cain and Cidlowski, 2017). It has been suggested that low concentrations of endogenous GC sensitize the innate immune system, while high concentrations of GC prevent excessive and/or prolonged immune responses (Cain and Cidlowski, 2017). In the same line, growing evidence implicates the immune system in stress resilience and coping through peripheral and central immune cells that act on the CNS, affecting, in turn, all stress-related neurobiological and neuroendocrine responses (Ménard et al., 2017). In addition, insufficient glucocorticoid-mediated regulation of stress hyper-responsiveness could result in impaired feedback regulation of relevant stress responses, especially immune activation/inflammation (Raison and Miller, 2003).

Therefore, the dysfunctional stress system neuroendocrine interface demonstrated in children and adolescents with ASD may be closely correlated to immunological alterations (Agorastos et al., 2019b). Altered immune responses have been reported in ASD ranging from alterations of peripheral immune markers to increased microglia activation in the CNS, all of them leading to a chronic state of low-grade inflammation (Onore et al., 2012; Hsiao, 2013; Barbosa et al., 2014). For example, several studies have demonstrated elevated plasma/serum pro-inflammatory cytokines, such as interleukin (IL)-1β, IL-6, IL-8, IL-12p40, IL-12, and interferon-γ (INF-γ) or a decreased production of cytokines that negatively regulate inflammation, such as TGFβ1 (Ashwood et al., 2008, 2011; Wei et al., 2013; Barbosa et al., 2014; Masi et al., 2015). Importantly, it has been shown that IL-6 stimulates the HPA axis at hypothalamic, pituitary, and adrenal level (Mastorakos et al., 1993; Stouthard et al., 1995; Crofford et al., 1997). Basal IL-6 is required for the sustained cortisol response to chronic stress, which is exerted through activation of the JAK/STAT3 signaling pathway; *vice versa*, cortisol exerts a mild inhibitory effect on the peripheral production of IL-6 (Papanicolaou et al., 1996; Girotti et al., 2013). On the other hand, catecholamines lead to an increase of plasma IL-6 (Søndergaard et al., 2000). Interestingly, upregulation of the JAK-STAT signaling pathway has been reported in ASD children, suggesting that immune pathways may play a certain role in the HPA axis dysfunction often demonstrated in ASD (Ahmad et al., 2017). Moreover, a recent study reported higher serum concentrations of high mobility group box 1 protein (HMGB1) in school-aged children with ASD than TD controls (Makris et al., 2021). HMGB-1 belongs in the alarmin family and is actively secreted under inflammatory conditions as an alarmin or late pro-inflammatory cytokine by different kinds of cells, including monocytes, tissue macrophages, astrocytes, and microglia (Fang et al., 2012). HMGB1 has been implicated in the stress-induced sensitization of innate immune cells and subsequent neuroinflammation (Zhang et al., 2019). Also, it has been suggested that, under stress, GC may induce HMGB1 release (Frank et al., 2015; Hisaoka-Nakashima et al., 2020).

Finally, it has been suggested that prolonged or chronic stress may lead to GR-mediated resistance in immune cells resulting in altered GC-related inflammation inhibitory signal (Avitsur et al., 2009; Cohen et al., 2012). Decreased GR mRNA levels and negative associations between cytokine mRNA and GR levels have been reported in ASD subjects, suggesting a possible role of inflammation in altered GR function in this neurodevelopmental disorder (Patel et al., 2016). Nevertheless, the GR function in ASD has not been sufficiently studied to date. Thus, more research is needed to better clarify the role of neuroimmunoendocrine pathways in the interplay between the immune and stress systems in ASD.

## Stress System Developmental Trajectory and the Role of Early Life Stress

A progressive divergence in the activity of the HPA axis and ANS following stress has been proposed to lead to the long-term impact of stress on the stress system and the chronic maintenance of PTSD symptoms in a large proportion (up to 20%) of subjects (Pervanidou, 2008). For example, in young individuals exposed to trauma, an initial increase in cortisol may be followed by a state of low cortisol production and progressively elevated norepinephrine concentrations as time passes from the traumatic event (Pervanidou et al., 2008). The first study that directly compared physiological responses to social evaluative threat (i.e., TSST) in adolescents vs. adults with ASD found that although there were no differences in baseline cortisol values, adults with ASD showed higher anticipatory circulating cortisol and sustained stress reactivity to a social evaluative threat, whereas the adolescent group had a more reactive cortisol response pattern with no anticipatory response (Taylor et al., 2018). In addition, it has been demonstrated that adolescents with ASD show a flatter diurnal slope, due to higher evening cortisol, than younger children with ASD (Muscatello and Corbett, 2018).

Early life stress has been recognized as a key mediator of developmental programming, since a limited insult during developmental vulnerability windows may increase the risk for either subclinical neuropsychological alterations or clinical conditions, such as neurodevelopmental disorders (Reynolds et al., 2013; Ghiani and Faundez, 2017; Pervanidou et al., 2017). It has been suggested that ELS, precisely prenatal maternal stress (PMS) and infant stress, is a risk factor for ASD (Beversdorf et al., 2018; Magariños et al., 2018). In fact, the embryo-fetal period and infancy are the most vulnerable periods of brain development (Kuhlman et al., 2017; Panisi et al., 2021). Maternal and fetal HPA axes, and the placenta are the most likely candidates regarding the mechanisms by which the effects of PMS might occur (Charil et al., 2010). Precisely, PMS may directly affect the HPA axis programming through alterations of the circulating GC levels and indirectly *via* alterations of fetal and maternal levels of cortisol and testosterone (Wilson et al., 2020). For example, PMS has been associated with higher basal GC secretion, stress reactivity alterations, and alterations in hippocampal GR in the offspring, potentially resulting in clinically significant negative influences on postnatal development (Charil et al., 2010). A growing literature suggests the association between PMS or/and postnatal environmental stressors and the risk for ASD in the offspring (Manzari et al., 2019). For example, maternal cortisol levels and maternal psychosocial stress have been associated with the trajectory of infant development influencing cognitive functioning at one year of age among healthy full-term infants, thus indicating that PMS plays a role in fetal programming (Davis and Sandman, 2010).

In addition, the outcomes of PMS have been considered sexually dimorphic, with females becoming more vulnerable to affective disorders, and males showing increased vulnerability to ASD (Davis and Pfaff, 2014). Furthermore, as it regards HPA axis programming, a shift from a hyper- to a hypo-responsive HPA axis may occur during the first 5 years of life (Pervanidou et al., 2020). A later, particularly sensitive developmental period is adolescence, when the HPA axis may transition from a period of decreased activity into a hyper-responsivity phase (Pervanidou et al., 2020). Thus, it has been suggested that sex- and age-related differences in the impact of ELS on stress system activity and reactivity may play a role in the risk of developing a specific mental disorder later in life. The largest population-based study to date showed that a stressful event, for example, the death of a first-degree relative, especially during the third trimester of pregnancy or during the second postnatal year, increased the risk for ASD in the offspring (Class et al., 2014). Also, mothers of ASD children reported a higher incidence of PMS at 21–32 weeks of gestation, with a peak at 25–28 weeks, than mothers of TD children (Beversdorf et al., 2005). Additionally, a prospective study showed that greater objective and subjective PMS resulting from a major natural disaster (i.e., the 1998 Quebec Ice Storm) predicted more severe autism-like traits in the offspring at age 6.5, although in the subclinical range (Walder et al., 2014). Of note, the moderating effects of the child’s biological sex were not significant and concerning the timing of ELS, objective stress significantly affected autistic traits in first-trimester exposed children (Walder et al., 2014).

Finally, ELS may contribute to the addition of epigenetic marks that affect the stress responses later in life (Agorastos et al., 2019b). In addition, there is accumulating evidence for the role of epigenetic alterations of specific genes in the pathophysiology of ASD (Loke et al., 2015; Dall’Aglio et al., 2018; Panisi et al., 2021). Therefore, epigenetic changes influenced by both genetic and environmental variation, and an interaction between the two, may play a central role in the long-term biological trajectories of ASD, including the disruption of developmental programming of stress-related structural and molecular neurobiological pathways.

## Discussion

This review provides a comprehensive overview of the current knowledge regarding the dysregulation of the two key stress system components (i.e., HPA axis and ANS) in ASD children and adolescents. Accumulating evidence suggests that children and adolescents with ASD may demonstrate dysfunction of the HPA axis and/or the ANS at baseline, in resting state, and during social and non-social stress challenges.

In summary, baseline and/or resting state cortisol dysregulation is not consistently found in children and adolescents with ASD. The majority of studies have shown comparable cortisol awaking response (CAR) between the ASD and TD groups (Zinke et al., 2010; Corbett and Schupp, 2014; Tomarken et al., 2015; Anesiadou et al., 2021). Also, lower diurnal salivary cortisol has been associated with higher rates of repetitive behaviors in children with ASD (Gabriels et al., 2013). Regarding cortisol circadian rhythm, a blunted diurnal slope from morning to evening, and higher evening cortisol values have been demonstrated in ASD than TD children (Corbett et al., 2009; Tomarken et al., 2015; Muscatello and Corbett, 2018). Importantly, pubertal development and age have been recognized as significant predictors of evening cortisol (Muscatello and Corbett, 2018). On the other hand, several studies have shown no differences between ASD and TD children in the diurnal decline and evening cortisol levels (Richdale and Prior, 1992; Marinović-Ćurin et al., 2008; Brosnan et al., 2009; Kidd et al., 2013; Anesiadou et al., 2021). The most consistent finding across studies is the higher between-subject and within-subject basal cortisol variability in children and adolescents with ASD compared to controls (Corbett et al., 2006, 2008, 2009; Kidd et al., 2013). Differences in HPA axis responsivity are more commonly found in ASD children and adolescents compared to controls and depend mainly on age and the nature of the stressor. Nevertheless, the findings are divergent and thus, they do not provide a definite conclusion regarding the direction (i.e., hyper- or hypo-responsivity) of HPA axis adaptation in ASD children and adolescents.

The majority of studies regarding resting state autonomic functioning converge on lower resting PNS activity accompanied by higher SNS activity in children and adolescents with ASD (Ming et al., 2005; Van Hecke et al., 2009; Bal et al., 2010; Neuhaus et al., 2014, 2016; Edmiston et al., 2016). In fact, higher baseline PNS activity has been associated with higher levels of social skills, more adaptive behaviors, better receptive language abilities, and less atypical attentional responsivity to sensory stimulation in children with ASD (Van Hecke et al., 2009; Bal et al., 2010; Patriquin et al., 2013; Condy et al., 2017; Lory et al., 2020). Accordingly, as it regards autonomic responsiveness of children and adolescents with ASD to several stressors, findings are indicative of sympathetic hyperarousal insufficiently attenuated by parasympathetic influences (Van Hecke et al., 2009; Bal et al., 2010; Kushki et al., 2013; Edmiston et al., 2016). In addition, convergent findings suggest that better skills and fewer difficulties are associated with sympathetic withdrawal and increased parasympathetic activity during social interaction (Neuhaus et al., 2016; Patriquin et al., 2019).

Discrepancies in the findings across studies may be due to several factors, such as differences in subject ages or developmental level, the severity of impairment, medication use, presence of comorbidities, the size of the sample groups, investigative methods employed, and the type of stressor used (Taylor and Corbett, 2014; Benevides and Lane, 2015). Thus, future research should focus on individual differences regarding the biobehavioral profile of ASD children and adolescents, taking into account the aforementioned confounding factors (Corbett et al., 2010, 2021; Schupp et al., 2013).

Importantly, the HPA axis and the ANS are interrelated components of an internal neurohormonal regulatory system (Thayer and Lane, 2000). The degree of HPA axis-ANS coordination in response to stress may be, at least partially, responsible for within-person differences regarding stress system function in children and adolescents with ASD (Chen et al., 2020). Therefore, attention should be paid to the interplay between the HPA axis and the ANS, which has a pivotal role in the regulation of stress system responses (Agorastos et al., 2019a; Chen et al., 2020). Future research may include a more thorough concurrent examination of both the HPA axis and ANS activity in ASD children and adolescents.

Moreover, several brain regions, such as the hippocampus, the amygdala, and the PFC play a pivotal role in the induction of neuroendocrine and behavioral stress responses (Fuchs and Flügge, 2003). These brain structures as part of the limbic system are suggested to play important roles in the regulation of the HPA axis (Pruessner et al., 2010). Thus, functional and/or structural alterations in these areas and/or altered functional connectivity between them may be responsible for stress system alterations seen in ASD children and adolescents (Schultz, 2005; Kennedy and Courchesne, 2008; Monk et al., 2010; Corbett et al., 2012; Rudie et al., 2012; Swartz et al., 2013; Pitskel et al., 2014; Von Dem Hagen et al., 2014; Ibrahim et al., 2019; Liu et al., 2020). For example, heightened activation of the stress system initially triggered by the amygdala may not be adequately inhibited by the PFC due to dysfunctional connectivity between these limbic structures in children and adolescents with ASD (Rudie et al., 2012). This may contribute to altered GC levels during the exposure to novel stimuli and lead to a prolonged stress response in dynamic social contexts in ASD. In addition, based on developmental factors, as personal experiences, the hippocampus may also become engaged in the activation of the limbic-HPA axis (Corbett et al., 2012). Thus, neuroendocrine alterations should be correlated with findings from concurrent imaging studies, especially regarding the functional connectivity between limbic structures that are influential in triggering the activation of the HPA axis. The use of functional and structural neuroimaging methods in combination with the concurrent assessment of stress hormones levels will allow investigating and understanding the neural processes associated with stress system dysregulation in ASD children and adolescents. This approach will provide new insights toward the understanding of stress perception mechanisms and variations of the stress response in children and adolescents with ASD.

Furthermore, the altered stress system neuroendocrine interface demonstrated in children and adolescents with ASD might be closely correlated to immunological alterations also seen in ASD (Ahmad et al., 2017; Makris et al., 2021). Also, inflammatory factors, such as cytokines, may play a role in altered GR function in this neurodevelopmental disorder (Patel et al., 2016). To date, there is a lack of studies that include assessments of the stress system baseline function or response to stressors at a hormonal level and concurrent investigation of immune dysfunction indicators in the same ASD subjects. Thus, future investigations of the stress system in ASD may assess the association of stress system dysregulation with immune abnormalities and investigate the possible role of altered GR function in children and adolescents with ASD (Frank et al., 2015; Masi et al., 2015; Patel et al., 2016). Such research endeavor may shed light on the possible role of altered interactions between immune and stress systems in the pathogenesis of ASD and may unravel possible pathological mechanisms that are involved in the mediation of ASD behavioral phenotype. Moreover, this approach may reveal molecular targets that are relevant to therapeutic aspects of ASD (Ahmad et al., 2017).

**FIGURE 1:**
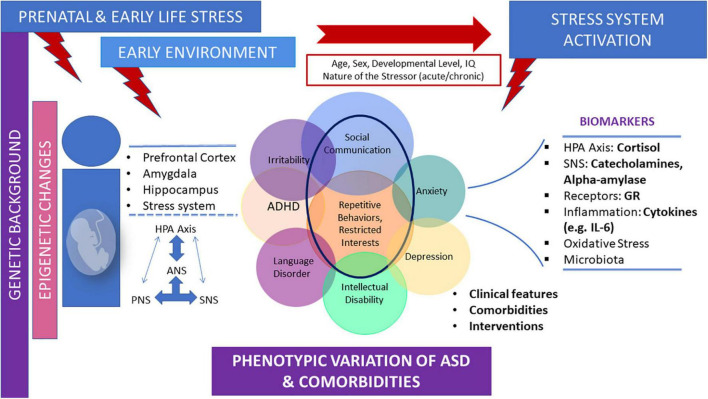
Parameters contributing to diverse neuroendocrine profiles and the main neurobiological mediators of the stress system in children and adolescents with ASD. The early environment, including prenatal and early life stress, which exerts its effects on a given genetic background, may influence the long-term biological trajectories of ASD mainly through the disruption of developmental programming of stress-related structural and molecular neurobiological pathways. These effects may lead to altered stress system activation, according to age, sex, developmental level, and the nature of the stressor. Stress system dysfunction in ASD children and adolescents is often demonstrated by alterations in several stress-related biomarkers and may be bidirectionally associated, at least in part, with phenotypic variations and comorbidities often shown in ASD.

Finally, prenatal stress and ELS are risk factors for ASD (Beversdorf et al., 2018; Magariños et al., 2018). Epigenetic alterations provoked by prenatal stress and/or ELS exposure may play a central role in the long-term biological trajectories of ASD, mainly through the disruption of developmental programming of stress related structural and molecular neurobiological pathways (Cattane et al., 2020; Panisi et al., 2021). Prospective studies are needed to determine the specific characteristics of the developmental trajectory of the stress system in children, adolescents, and adults with ASD, and to further examine the role of ELS in the long-term biological trajectories of ASD. Accumulating evidence underlies the association of ELS exposure and the risk for ASD in the offspring. However, there are some inconsistencies in the literature. For example, a study found no association between PMS and ASD risk in the offspring (Rai et al., 2012). In addition, although it has been suggested that PMS may be associated with ASD in the offspring, there is no consensus as to the critical gestational periods (Manzari et al., 2019). Carefully controlled prospective studies are needed to investigate the causal processes and mechanisms that may underlie the association of ELS and ASD. Figure 1 depicts the parameters contributing to diverse neuroendocrine findings regarding stress system dysfunction observed in children and adolescents with ASD, as well as the main neurobiological mediators of the stress system in ASD (Figure 1).

To date, the presence of inconsistencies in the observations regarding the stress system dysregulation in children and adolescents with ASD is the main caveat to any generalizations and biomarkers detection. Also, the fact that the majority of data points toward inconclusive results raises a question about the supposed specific role of the stress system in the pathophysiology and the clinical presence of ASD. While there is evidence of an aberrant cortisol circadian rhythm and alterations in stress system responsivity in children and adolescents with ASD, the extent and the relations of these changes and their underlying mechanisms remain unclear and warrant further study. Thus, the first step needed, to clarify the role of at least specific aspects of stress system alterations in ASD, is the in-depth research toward the understanding of possible mechanisms linking stress system alterations with this neurodevelopmental disorder. The elimination, where is possible, and the thorough study of any confounding factors, such as the severity of symptoms, comorbidities, developmental level, and gender are also necessary steps. Moreover, large, prospective, and methodologically focused studies in well-characterized clinical samples are needed, to clarify if the stress system is involved in ASD in a specific manner and/or if etiologic relations between stress system dysregulation and neurodevelopmental deficits exist. Therefore, the question of whether the associations between stress system alterations and ASD symptoms are bidirectional remains. The current review is an attempt to foster a multimodal approach to the stress system dysregulation found in ASD children and adolescents. An integrated approach should comprise the assessment of possible correlations between neuroendocrine alterations and imaging findings, immunological alterations, epigenetic marks, and prenatal and early life environmental risk factors that play a pivotal role in the stress system programming. Also, it is necessary to comprehensively investigate the functional importance of the circadian clock in neurodevelopment and its dysregulation in neurodevelopmental disorders (Lorsung et al., 2021). Finally, as to the question of the specificity of the observations regarding stress system dysregulation in ASD, it would be elucidating if future studies investigated the role of the stress system in the autism-like traits found in the general population. Beyond doubt, the continued growth in understanding of downstream mechanisms will result in an increased ability to identify those individuals at greatest risk for developing ASD and also multiple potential points for prevention or early intervention.

## Author Contributions

PP: conceptualization and supervision. GM: writing and original draft preparation. AA, GC, and PP: writing, reviewing, and editing. All authors have read and agreed to the published version of the manuscript.

## Conflict of Interest

The authors declare that the research was conducted in the absence of any commercial or financial relationships that could be construed as a potential conflict of interest.

## Publisher’s Note

All claims expressed in this article are solely those of the authors and do not necessarily represent those of their affiliated organizations, or those of the publisher, the editors and the reviewers. Any product that may be evaluated in this article, or claim that may be made by its manufacturer, is not guaranteed or endorsed by the publisher.

## Abbreviations

ASD: autism spectrum disorder
TD: typically developing children
HPA: hypothalamic–pituitary–adrenal axis
CNS: central nervous system
ANS: autonomic nervous system
SNS: sympathetic nervous system
PNS: parasympathetic nervous system
CRH: corticotropin-releasing hormone
ACTH: adrenocorticotropin hormone
GC: glucocorticoids
GR: glucocorticoid receptors
CAR: cortisol awakening response
sAA: salivary alpha-amylase
EDA: electrodermal activity
RSA: respiratory sinus arrhythmia
HRV: heart rate variability
ELS: early life stress
PMS: prenatal maternal stress.
